# Transparent metafilms for enhanced thermal regulation in energy-efficient windows

**DOI:** 10.1515/nanoph-2025-0351

**Published:** 2025-09-29

**Authors:** Biyuan Wu, Yue Ren, Xiqiao Huang, Meijie Chen, Yong Li, Jiangtao Li, Yang Kou, Xiaohu Wu

**Affiliations:** Thermal Science Research Center, Shandong Institute of Advanced Technology, Jinan, 250100, Shandong, P.R. China; School of Power and Energy, Northwestern Polytechnical University, Xi’an, 710072, Shaanxi, P.R. China; State Key Laboratory of Cryogenic Science and Technology, Technical Institute of Physics and Chemistry, Chinese Academy of Sciences, Beijing, 100190, P.R. China; University of Chinese Academy of Sciences, Beijing, 100049, P.R. China; School of Energy Science and Engineering, Central South University, Changsha, 430001, Hunan, P.R. China; Research Center for Laser Physics and Technology, Technical Institute of Physics and Chemistry, CAS, Beijing, 100080, P.R. China

**Keywords:** transparent metafilms, energy-efficient window, radiative cooling, spectrally selective regulation

## Abstract

Transparent metafilms with spectrally selective properties have shown great potential in energy-efficient window systems. Most previous studies focused on optimizing materials and thicknesses to enhance visible transmittance and near-infrared (NIR) reflectance. However, few have considered how the position of the metafilms on the glass affects overall optical and thermal performance, especially in the mid-infrared (MIR) range critical for radiative cooling. In this work, we propose and analyze a five-layer TiO_2_/Ag/TiO_2_/Ag/TiO_2_ structure and systematically evaluate its performance under two typical installation scenarios. Numerical simulations based on the transfer matrix method show that both configurations maintain a high visible transmittance (∼0.88) and an effective NIR reflectance (∼0.98). Notably, a substantial difference is observed within the atmospheric transparency window 8–14 μm, where the interior-coated configuration possesses a high emissivity of 0.8. This value significantly exceeds the average emissivity of 0.01 found for the exterior-coated configuration, thereby resulting in superior passive radiative cooling capability. Moreover, we also compared the net radiative cooling power under the two configurations. These findings reveal that the position of the transparent metafilms critically influences MIR radiation. Coating placement on the interior surface not only maintains favorable solar modulation but also markedly enhances the thermal dissipation. This study offers theoretical guidance and practical insight into the design and implementation of metafilms in energy saving window systems aimed at reducing energy consumption, especially in regions with hot climates.

## 1 Introduction

With the continuous growth of global energy consumption and the intensification of climate change, enhancing the building energy efficiency has become a crucial pathway to achieving energy saving and emission reduction goals [1], [2], [3]. As a key component of building envelopes, windows not only affect indoor lighting but also play a vital role in the thermal management of buildings. Therefore, the development of energy-efficient window systems with selective spectral regulation has become a central focus of building energy-saving technologies [4].

To meet daylighting requirements while reducing cooling loads, energy-saving windows must achieve high transmittance in the visible region and simultaneously block near-infrared (NIR) radiation, which accounts for over 50 % of solar energy [5], [6]. Transparent heat regulating coatings, which combine high visible transparency with strong NIR reflectance, have attracted growing attention in energy-efficient windows and smart glass applications [7], [8], [9], [10]. Among various transparent heat regulating coatings technologies, metal/dielectric multilayer films stand out due to their simple structure and excellent optical properties [11], [12], [13], [14], [15], [16]. Typical configurations, such as dielectric/metal/dielectric (DMD) stacks, achieve effective regulation of the solar spectrum in the 0.3–2.5 μm wavelength range by optimizing the materials and thicknesses of each layer, thus reducing solar heat gain while ensuring adequate daylighting [17], [18], [19], [20], [21]. Representative examples include ZnO/Ag/ZnO [22], WO_3_/Ag/WO_3_ [23], TiO_2_/Ag/TiO_2_ [24], [25], [26], [27], and WO_3_/Au/WO_3_ [28]. However, existing studies primarily focus on optimizing the structural parameters of the coatings, while systematic investigations into the impact of their placement (interior vs. exterior) on the glass surface on optothermal performance remain limited.

In addition to visible and NIR regulation, thermal radiation control in the mid-infrared (MIR: 2.5–25 μm) range, especially within the atmospheric transparency window (8–14 μm), is also critically important for building thermal management [29], [30], [31], [32], [33], [34]. For instance, Zhou et al. developed energy-saving windows with radiative cooling functionality by adding a top emissive layer to NIR reflective structure [35]. Conventional glass inherently exhibits high emissivity in the MIR range, enabling a certain degree of passive radiative cooling under suitable conditions. However, the strong reflectance of the metal layer can block the glass’s infrared emission to the external environment when transparent heat regulating coatings are installed on the exterior side of glass, thereby weakening or even suppressing its heat dissipation capability. This phenomenon has not been sufficiently considered in current energy-saving window designs.

In this paper, we design a transparent metafilm composed of five-layer TiO_2_/Ag/TiO_2_/Ag/TiO_2_ structure and systematically study its spectral response characteristics when applied to either the interior or exterior side of glass. The transmittance, reflectance, and emittance characteristics of the structure are systematically analyzed across the visible, NIR, and MIR spectral ranges. Based on the emissivity spectrum within the MIR atmospheric window, the radiative cooling power of the two configurations is further calculated at an ambient temperature of 303.15 K. Furthermore, indoor and outdoor thermal experiments demonstrate the temperature regulation capability of the transparent metafilm under two cases. This work highlights the significant influence of the installation position on the overall performance of the transparent metafilm, offering practical insights for enhancing building energy efficiency and indoor thermal comfort.

## 2 Model and method

**Figure 1:**
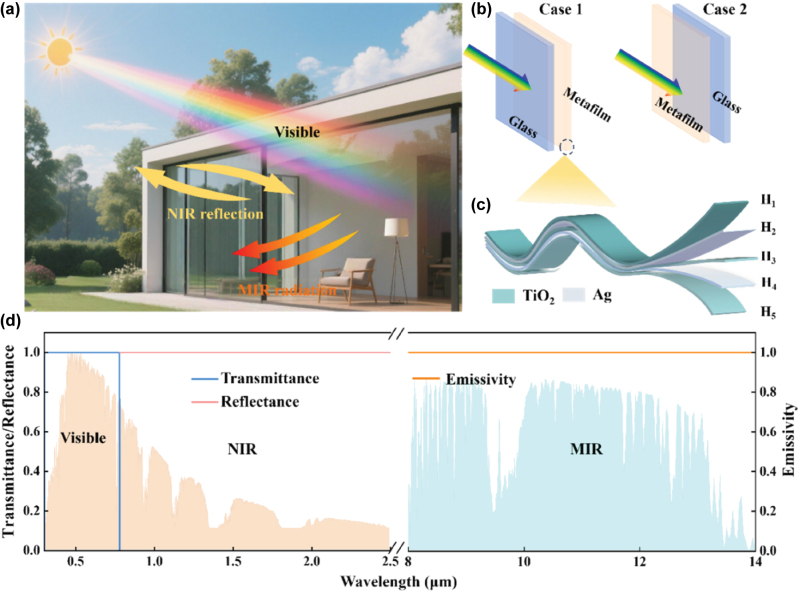
Schematic diagram of transparent metafilm structure and ideal spectrum for energy-efficient windows. (a) Schematic illustration of solar spectrum-selective modulation by the metafilm. (b) Structural configurations of the transparent metafilm applied on the inner side (Case 1) and the outer side (Case 2) of glass. (c) Structural diagram of the transparent metafilm, composed of a TiO_2_/Ag/TiO_2_/Ag/TiO_2_ multilayer. (d) Ideal spectral response of an energy-efficient window.

As illustrated in Figure 1a, an ideal architectural window should possess both excellent optical transparency and effective thermal regulation capability. In the evaluation of ultrathin glass performance, visible light transmittance is a critical parameter, as optical transparency is a fundamental functional requirement of windows that directly influences human visual perception of the external environment. In residential and office settings, there is a general expectation for windows to exhibit high visible transmittance to ensure sufficient daylighting and clear exterior views. Inadequate transmittance can lead to insufficient indoor illumination and reduced visibility of outdoor scenes, ultimately compromising visual comfort. The thermal regulation performance of architectural glazing can be achieved through enhanced reflectance in the NIR region and improved emissions in the atmospheric window (AW) band. The NIR region contains a significant portion of solar thermal energy, and excessive transmission under intense sunlight can cause indoor overheating and thermal discomfort. Therefore, increasing the reflectance in the NIR region effectively blocks the incoming solar heat. In contrast, enhancing emissivity in the AW band facilitates passive radiative cooling, which contributes to temperature reduction by dissipating heat into outer space. Thus, the spectral characteristics of an energy-efficient window should align with the ideal profile illustrated in Figure 1d, featuring high transmittance in the visible range, high reflectance in the NIR region, and high emissivity within the AW region.

To achieve the desired spectral regulation, a transparent metafilm with a five-layer dielectric/metal alternating structure (D/M/D/M/D) is designed, comprising TiO_2_/Ag/TiO_2_/Ag/TiO_2_ layers, as illustrated in Figure 1c. The permittivity of Ag is described by the Drude model [36], while the permittivity of TiO_2_ is obtained from Ref. [37]. Furthermore, as illustrated in Figure 1b, this work investigates the impact of two representative installation configurations on the comprehensive optical-thermal performance of the window system. In Case 1, the metafilm is applied to the inner surface of the glass, leaving the exterior glass exposed. In Case 2, the metafilm is deposited on the outer surface, directly interacting with the incident solar radiation. To evaluate the spectral performance of the two installation configurations, numerical simulations are conducted using the transfer matrix method (TMM) [38], [39]. This method based on Maxwell’s equations solves the propagation of electromagnetic waves through multilayer structures and enables the calculation of reflectance (R) and transmittance (T) at arbitrary wavelengths. Since all materials used in this study are reciprocal, the emissivity spectrum can be derived from the absorptance, calculated as *A* = 1 − *R* − *T*, where *A* denotes absorptance.

## 3 Results and discussion

**Figure 2:**
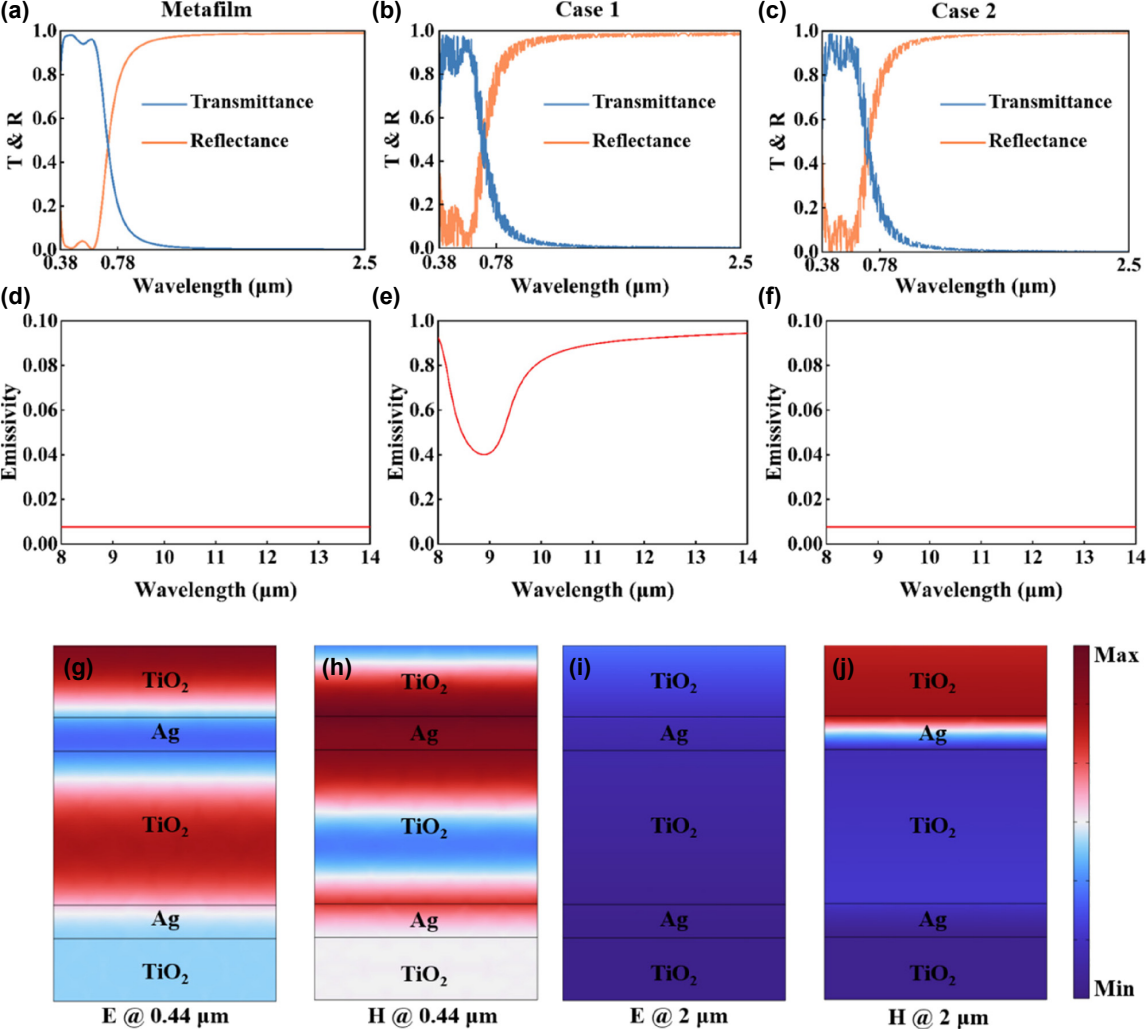
The spectra and electromagnetic field distributions of the proposed structure. (a–c) Transmittance, reflectance, and (e, f) emissivity spectra of (a, d) the transparent metafilm, (b, e) Case 1, and (c, f) Case 2, respectively. (g) Electric field and (h) magnetic field distributions at 0.44 μm; (i) electric field and (j) magnetic field distributions at 2 μm.

By applying the TMM in conjunction with parameter sweeping, the optimal thicknesses of the five-layer structure are determined to be 32 nm, 15 nm, 69 nm, 15 nm, and 28 nm for layers *H*_1_ through *H*_5_, respectively. To validate the spectral modulation capability of the designed structure, the optical properties of the freestanding metafilm are first simulated, including transmittance, reflectance, and emissivity, as shown in Figure 2a and d. The results indicate that the metafilm exhibits a high average transmittance of over 0.95 in the visible range (0.38–0.78 μm), fulfilling the requirements for natural daylighting. Meanwhile, in the NIR range (0.78–2.5 μm), it demonstrates excellent reflectance, exceeding 0.97, which is effective for blocking solar heat gain. However, the emissivity remains near zero in the AW region, indicating negligible radiative cooling capability from the coating itself. Overall, this multilayer structure achieves selective spectral regulation of solar radiation, making it a promising candidate for energy-efficient window applications.

To further evaluate the impact of coating placement on the overall optical and thermal performance of the window system, two configurations are compared, in which the metafilm was applied to either the interior (Case 1) or exterior (Case 2) surface of a ∼1 mm thick glass substrate. For Case 1 (Figure 2b and e), the average transmittance in the visible range is approximately 0.89, and the reflectance in the NIR region is around 0.99. Notably, the emissivity in the AW significantly increases, reaching values above 0.8, which enables effective MIR radiative cooling by leveraging the intrinsic high emissivity of the glass. In contrast, Case 2 maintains strong optical performance in the visible and NIR regions, with a visible transmittance of approximately 0.89 and NIR reflectance of around 0.98, demonstrating effective solar modulation. However, since the metafilm is directly exposed to the environment, it exhibits strong reflection in the MIR region, thereby suppressing the radiative cooling capability of the glass substrate.

Figure 2g–j shows the electromagnetic field distributions of the metafilm at representative wavelengths to illustrate the underlying physical mechanism of the spectral modulation. At a wavelength of 0.44 μm, the electric field is mainly localized in the top two TiO_2_ layers, while the magnetic field is concentrated in the Ag layers. The spatial anti-phase distribution of the electric and magnetic fields satisfies the standing wave condition, which explains the high transmittance in the visible region. In contrast, at a wavelength of 2 μm, the electric field intensity becomes weaker, and the magnetic field is predominantly localized in the upper part of the structure, revealing the physical origin of the high NIR reflectance.

**Figure 3:**
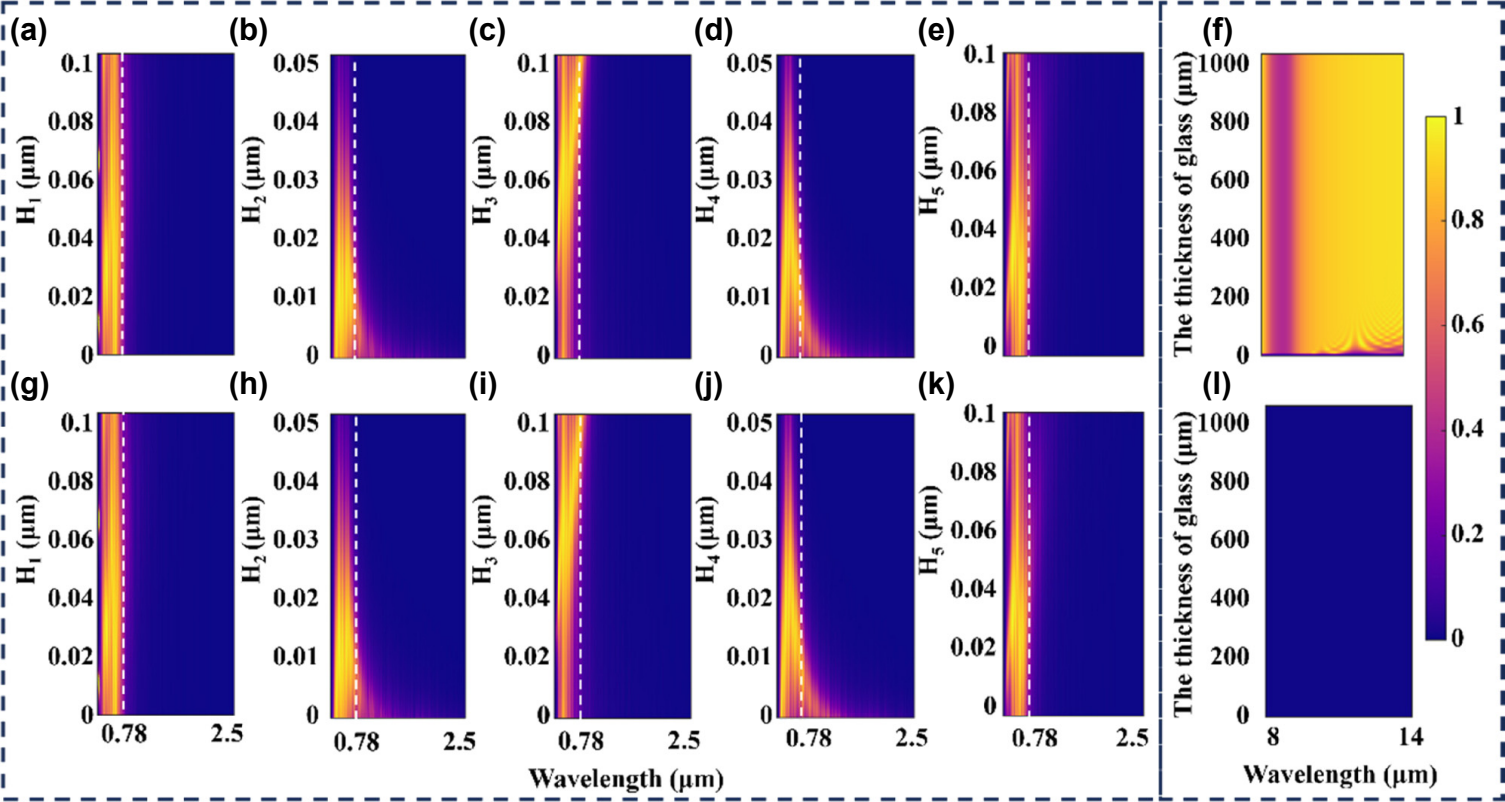
Influence of the thickness on the spectral response. (a–e) and (g–k) show the variation of transmittance in the visible to NIR range as a function of individual layer thicknesses for Case 1 and Case 2, respectively. (f) and (l) present the emissivity within the atmospheric transparency window as a function of the glass (SiO_2_) thickness for Case 1 and Case 2, respectively.

The thickness of each layer in the structure plays a crucial role in determining the overall device performance. Figure 3 systematically investigates the thickness dependence of the energy-saving window under two structural configurations. Figure 3a–e and g–k presents the effects of varying parameters *H*_1_ to *H*_5_ on the transmittance spectra. Overall, both configurations exhibit similar trends. Within the investigated thickness ranges, the structures maintain high transmittance in the visible region while effectively suppressing transmission in the NIR region. Specifically, when *H*_1_ ranges from 0.02 to 0.04 μm, the visible transmittance approaches unity. When *H*_2_ and *H*_4_ are below 0.01 μm, a noticeable portion of NIR radiation can penetrate the structure, which compromises the thermal insulation performance. In contrast, variations in *H*_3_ and *H*_5_ produce only minor effects within the explored parameter space, thereby validating the parameter choices made in Figure 2. Figure 3f and l illustrates the emissivity as a function of glass thickness in the AW for Case 1 and Case 2, respectively. When the proposed metafilm is placed on the inner side of the glass (Case 1), the structure consistently maintains high MIR emissivity. In contrast, when the metafilm is located on the outer side (Case 2), the emissivity remains nearly zero regardless of the thickness of the glass. These results indicate that the thickness of the glass does not significantly affect the thermal emission capability of the window. Instead, the emissive behavior is primarily governed by the configuration of the structure.

**Figure 4:**
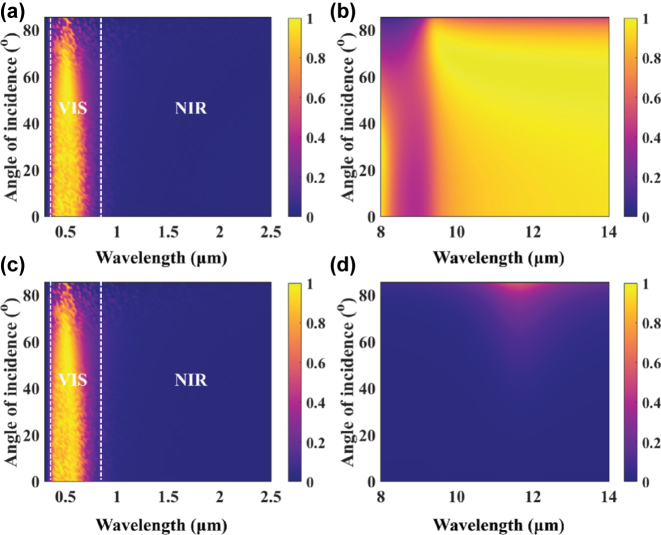
Influence of the incident angle on the (a, c) transmittance and (b, d) emissivity spectra for (a, b) Case 1 and (c, d) Case 2, respectively.

For energy-efficient window systems, it is essential not only to achieve excellent optical and thermal regulation under normal incidence but also to maintain stable spectral performance under oblique incidence. This is particularly important for adapting to the dynamic solar illumination conditions in real architectural environments, where the incident angle varies with time and orientation. Figure 4 presents the angular dependence of the transmittance and emissivity spectra for the metafilm applied on the interior surface (Case 1) and the exterior surface (Case 2) of the glass. As shown in Figure 4a and c, both configurations maintain high transmittance in the visible range when the incident angle is less than 60°, ensuring sufficient daylighting. With further increases in the incident angle, transmittance slightly decreases. Meanwhile, the NIR transmittance remains close to zero across all angles, demonstrating strong solar heat rejection performance even under slanted sunlight. Figure 4b and d shows the angular dependence of emissivity in the AW region. For Case 1, the emissivity remains high and nearly invariant with the incident angle, indicating excellent directional stability in radiative cooling capability. This suggests that the structure can continuously emit thermal radiation into the sky throughout the day, thereby effectively lowering indoor temperature during hot seasons and improving thermal comfort. In contrast, Case 2 shows consistently low emissivity in the same spectral range, indicating that the structure significantly suppresses the intrinsic thermal emission of the glass, thereby limiting the effectiveness of radiative heat dissipation.

To assess the thermal performance of energy-efficient windows under the two configurations, the temperature of the radiative cooler is modeled by solving the steady-state heat balance equation. In this analysis, we consider a radiator with surface area *A* and temperature *T*, oriented in such a way that its surface normal points toward the zenith. Under these assumptions, the net radiative cooling power *P*_net_ can be expressed as [40]

(1) $$P_{\text{net}}\left(T\right)=P_{\text{ard}}\left(T\right)-P_{\text{c}}$$

Here, *P*_ard_ denotes the radiative thermal load of the emitter resulting from radiative energy exchange with the surrounding environment, which can be calculated by

(2) $$P_{\text{ard}}=P_{\text{emi}}\left(T\right)-P_{\text{atm}}\left(T_{\text{amb}}\right)-P_{\mathrm{sun}}\left(T\right)$$

where *P*_emi_(*T*) is the energy emitted via radiator, which can be formulated as

(3) $$P_{\text{emi}}\left(T\right)=A\int d\Omega \cos\theta \overset{\infty }{\underset{0}{\int }}d\lambda I_{BB}\left(T,\lambda \right)\varepsilon \left(\lambda ,\theta \right)$$

In this context, Ω denotes the solid angle, *θ* is defined as the angle between the direction of Ω and the surface normal, *ε*(*λ*, *θ*) represents the emissivity of the material at a given wavelength *λ* and angle *θ*, and *I*_BB_(*T*, *λ*) corresponds to the spectral irradiance of a blackbody at temperature *T* and wavelength *λ*. In Eq. (2), *P*_atm_(*T*_amb_) is the portion of the downward atmospheric radiation absorbed by the radiator, which can be expressed as

(4) $$\begin{array}{ll} P_{\text{atm}}\left(T_{\text{amb}}\right) & =A\int \text{d}\Omega \cos\theta \overset{\infty }{\underset{0}{\int }}\text{d}\lambda {\text{I}}_{\text{BB}}\left(T_{\text{amb}},\lambda \right)\\ & \;\times \varepsilon_{\text{atm}}\left(\lambda ,\theta \right)\varepsilon \left(\lambda ,\theta \right) \end{array}$$

where *ɛ*_atm_(*λ*, *θ*) = 1 − *t*(*λ*)^1/cos*θ*^ is the emissivity of the atmosphere. *t*(*λ*) is the transmission coefficient of the atmosphere in the zenith direction. Assuming that *I*_sun_(*λ*) denotes the AM 1.5 solar spectrum and *θ*_sun_ represents the incident angle of solar radiation, the absorbed solar power by the radiative cooler can be formulated as

(5) $$P_{\mathrm{sun}}\left(T\right)=A\cos\theta_{\text{sun}}\overset{\infty }{\underset{0}{\int }}\text{d}\lambda I_{\text{sun}}\left(\lambda \right)\varepsilon \left(\lambda ,\theta_{\text{sun}}\right)$$

In Eq. (1), the last term *P*_c_ represents the heat load resulting from conductive and convective heat exchange between the radiator and the environment, which can be expressed as

(6) $$P_{\text{c}}=Ah\left(T_{\text{amb}}-T\right)$$

where *h* denotes the effective non-radiative heat transfer coefficient that quantifies the conductive and convective heat exchange described above.

**Figure 5:**
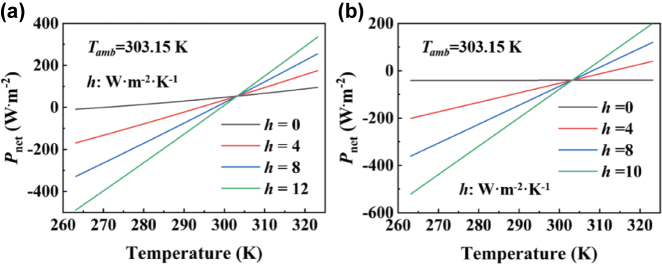
The radiant power as a function of the temperature at different *h* for (a) Case 1 and (b) Case 2, respectively.

Based on the above equations, we present in Figure 5 the variations of the net cooling power *P*_net_ as a function of temperature *T* under two different scenarios. Assuming an ambient temperature of *T*_amb_ = 303.15 K, we investigate the cooling performance under different heat transfer coefficients *h*, where *h* = 0 represents an ideal thermally insulated condition. For Case 1 (Figure 5a), the window enters a radiative cooling mode when the temperature exceeds approximately 270 K in the absence of conductive and convective heat transfer. As the temperature increases, the net radiative cooling power rises steadily, primarily due to the high emissivity in the MIR range. When the window temperature approaches the ambient temperature 303.15 K, the net cooling power reaches 54.43 W/m^2^. This positive heat flux (*P*_net_>0) indicates that the window is effectively radiating heat to the surrounding environment. In contrast, Figure 5b shows the results for Case 2. When the window temperature equals the ambient temperature, the net cooling power is −40.23 W/m^2^. This indicates that the window is absorbing energy from the environment rather than radiating heat, placing the system in a passive heating state.

**Figure 6:**
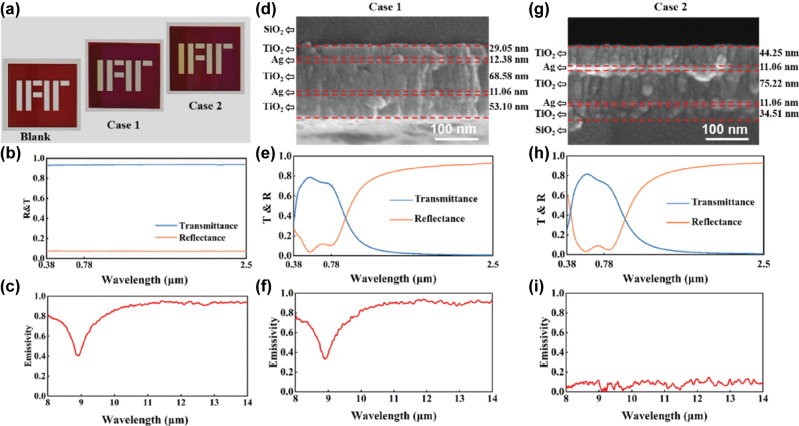
Sample appearance and experimental results. (a) The appearances of the samples under the natural light, from left to right: blank glass, Case 1, and Case 2. The experimentally measured (b) transmittance and reflectance spectra and (c) emissivity of the blank glass. The cross-sectional SEM images of (d) Case 1 and (g) Case 2, respectively. The experimentally measured transmittance and reflectance spectra of (e) Case 1 and (h) Case 2, as well as emissivity spectra of (f) Case 1 and (i) Case 2.

Next, the performance of the designed structure is experimentally validated. The five-layer multilayer structure is deposited on a 1 mm-thick glass (SiO_2_) substrate using magnetron sputtering at room temperature. Prior to deposition, the glass substrate was cleaned with acetone in an ultrasonic bath, followed by sequential rinsing with ethanol and deionized water to eliminate organic contaminants. The treated substrate was then loaded into the deposition chamber, which was evacuated to a base pressure of 2.0 × 10^−5^ Pa using a cryogenic vacuum pump. High-purity (99.99 %) Ag and TiO_2_ targets were employed for sputtering. The deposition rates and film thickness were monitored and controlled in real-time using a white-light interferometer. The Ag and TiO_2_ layers were deposited at rates of 0.5 nm/s and 0.06 nm/s, respectively. Figure 6a presents the visual appearance of the bare glass (labeled as “Blank”), Case 1, and Case 2 under natural light, respectively. It is evident that the samples exhibit high transparency in the visible wavelength range. The surface morphology of the thin films was examined by scanning electron microscopy (Sigma 300, ZEISS). Figure 6d and g shows the cross-sectional scanning electron microscope image of the sample, clearly revealing the six-layer structure composed of the multilayer coating and the glass. For Case 1, the thicknesses of the TiO_2_/Ag multilayer stack are measured to be 29.05 nm, 12.38 nm, 68.58 nm, 11.06 nm, and 53.10 nm, respectively. In contrast, the layer thicknesses in Case 2 are 44.25 nm, 11.06 nm, 75.22 nm, 11.06 nm, and 34.51 nm, respectively. The optical properties of the multilayer films in the UV–vis–NIR range were characterized using UV–vis–NIR spectroscopy (Cary 7000, Agilent). Fourier-transform infrared spectroscopy (Invenio S, Bruker) was employed to evaluate the optical performance of the films in the MIR region. First, the spectral response and thermal radiative performance of the blank glass were measured, as shown in Figure 6b and c. It can be observed that the glass consistently exhibits high transmittance and low reflectance in the visible and NIR region. In the atmospheric window range, except for a local emissivity dip around 9 μm, the glass maintains high emissivity across the remaining wavelengths. Figure 6e presents the experimentally measured transmittance and reflectance spectra of the Case 1 sample. The transmittance and reflectance curves closely follow the trend predicted by theoretical calculations. Figure 6f shows the emissivity in the MIR range, which agrees with the theoretical simulation. For Case 2, the experimental results are shown in Figure 6h and i. The sample exhibits high transmittance in the visible range, strong reflectance in the NIR region, and nearly zero emissivity in the MIR band. These characteristics demonstrate excellent agreement with theoretical design. To more clearly illustrate the performance of different configurations, Table 1 presents the visible transmittance, NIR reflectance, and atmospheric window emissivity for blank glass, Case 1, and Case 2. Specifically, the average transmittance of Case 1 and Case 2 is 0.77 and 0.79, respectively. Compared with blank glass, Case 1 and Case 2 present a slightly reduced visible transmittance while still meeting the requirements for indoor lighting. The NIR reflectance is 0.78 for Case 1 and 0.74 for Case 2, indicating that both samples exhibit high visible transmittance and NIR reflectance. In terms of MIR emissivity, Case 1 reaches 0.84, which is significantly higher than 0.08 for Case 2. These results further confirm the optical and radiative performance of the samples.

**Table 1:** Average transmittance, reflectance, and MIR emissivity for blank glass, Case 1, and Case 2.

Structure	*T*_VIS_	*R*_NIR_	*ɛ*_MIR_
Blank glass	0.93	0.07	0.80
Case 1	0.77	0.78	0.84
Case 2	0.79	0.74	0.08

**Figure 7:**
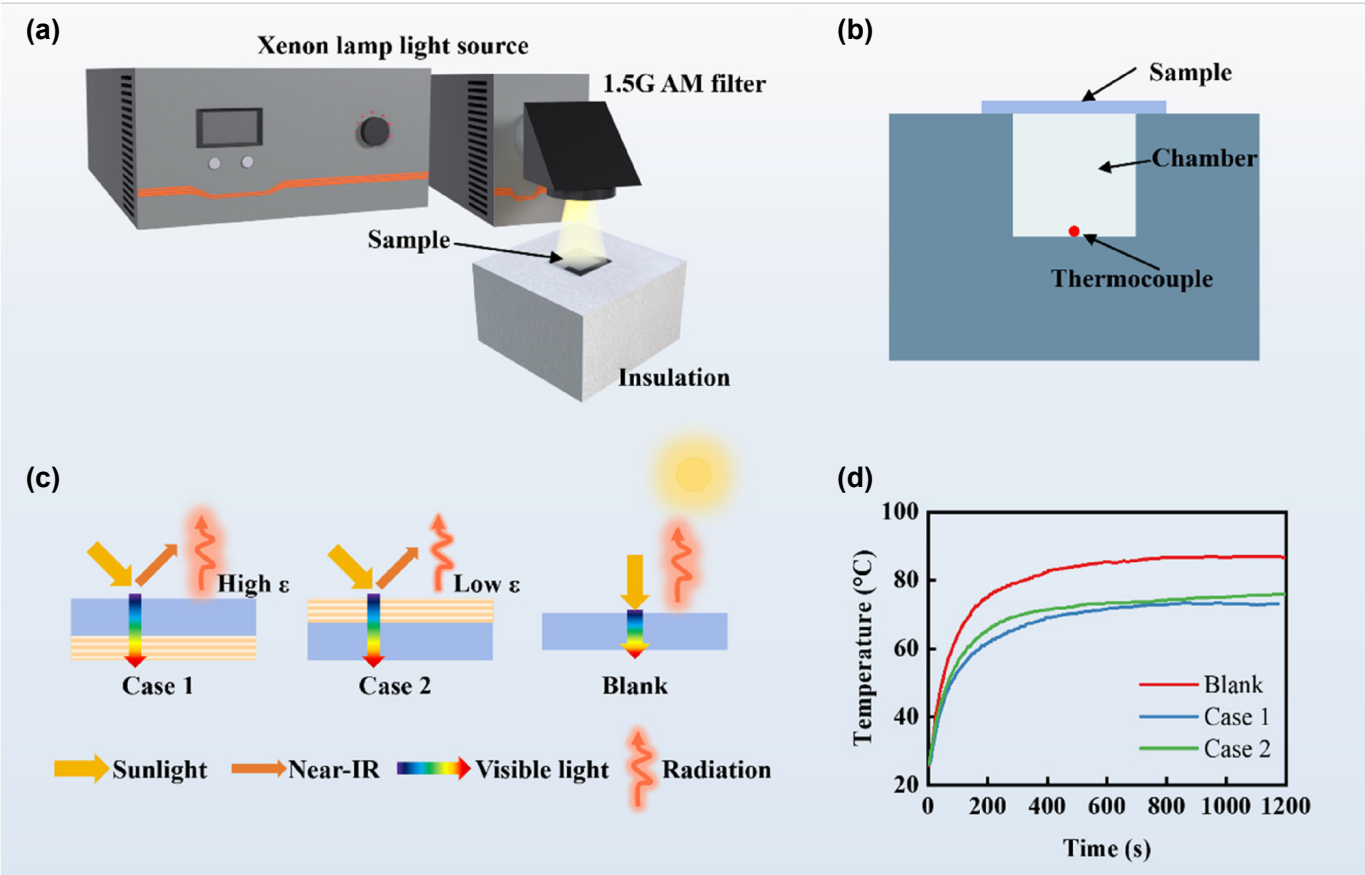
Schematic diagram of the indoor experimental system and corresponding test results. (a) Schematic diagram of the experimental system for measuring the window’s temperature response. (b) Schematic illustration of the absorption chamber. (c) Light propagation paths of three samples. (d) Measured variation of inner temperature over time.

The indoor thermal experiment was conducted to evaluate the temperature-regulating capability of the coatings. Three samples (blank, Case 1, and Case 2) were sequentially placed on the roofs of confined rooms with black inner walls. As shown in Figure 7a, a xenon lamp was used to simulate solar irradiation. Each absorption chamber has an internal space of 2 cm × 2 cm × 2 cm, constructed from a 4-cm-thick foam board lined with black tape on the inside and covered with silver aluminum foil on the outside. The top of the devices was covered in turn with the three samples (Blank, Case 1, and Case 2). The temperatures inside the chambers and in the ambient environment were measured using thermocouples (Figure 7b). All data were recorded in real time and transmitted to a computer via a data collector. Due to the solar heating by the transmitted solar radiation, the inner temperatures of these cases are higher than the ambient temperature $\bar{T}\text{amb}$ = 25 °C. As shown in Figure 7c, when the incident light strikes the glass surface, Case 1 and Case 2 transmit visible light while reflecting NIR radiation, thereby reducing heat absorption inside the chamber. In contrast, the blank glass transmits both visible and NIR wavelengths completely, resulting in an increase in the internal temperature. As illustrated in Figure 7d, the steady-state temperatures of the blank, Case 1, and Case 2 are 86.9, 73.1, and 75.9 °C, respectively. Compared with bare glass, Case 2 can achieve a temperature drop of 11.0 °C due to its high solar reflectance, while the thermal emittance on the outer side is low. Further, by enhancing the thermal emittance, Case 1 can achieve a temperature drop of 2.8 °C compared with Case 2. These results indicate that the designed Case 1 can efficiently decrease the room temperature, which is better than Case 2. This makes the installation position of the transparent metafilm a decisive factor in achieving optimal thermal performance.

**Figure 8:**
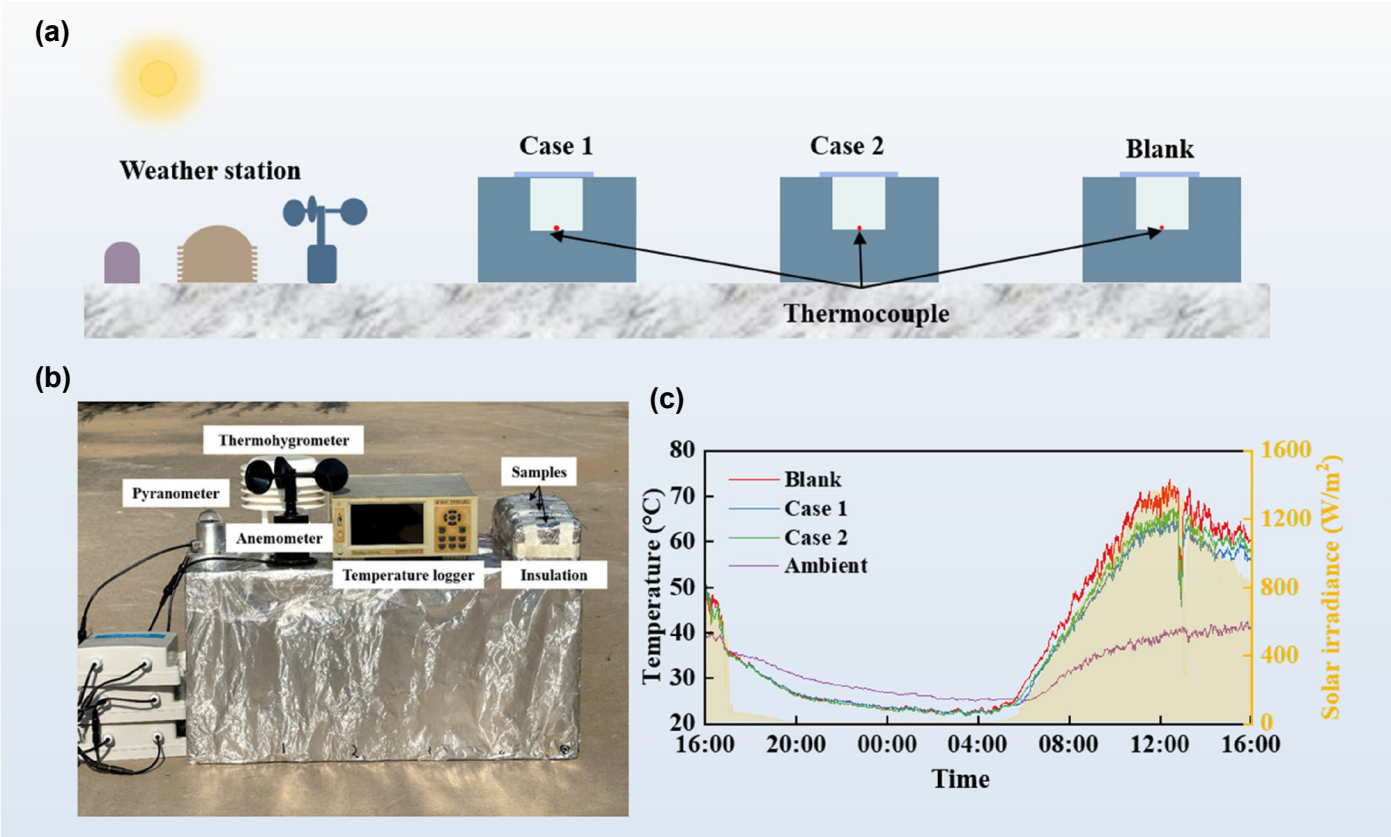
Schematic diagram of the outdoor experimental system and corresponding test results. (a) Schematic illustration and (b) photograph of the outdoor experiment. (c) Temperature variation in the experiment from 16:00 p.m., July 17, 2025, to 16:00 p.m., July 18, 2025. The yellow-shaded area shows the measured solar irradiance.

Furthermore, the outdoor experiment was conducted on a flat roof in Langfang, China, on July 18, 2025. The internal space of absorption chambers is 2 cm × 2 cm × 2 cm, which is made of a 4-cm-thick foam board with black tape inside and silver aluminum foil outside, as shown in Figure 8a and b. The top of the three devices is covered with Case 1, Case 2, and blank glass in turn. A meteorological workstation was set up to measure outdoor wind speed, temperature/humidity, and solar irradiance using an anemometer, a thermohygrometer, and a pyranometer, respectively. The internal temperature of the chamber was monitored using thermocouples. All data are recorded in real-time and transferred to the computer via the data collector. The continuous 24-h outdoor measurement was conducted for the three samples (blank glass, Case 1, and Case 2) to evaluate both daytime and nighttime cooling performance. As shown in Figure 8c, under strong solar irradiation during the daytime (10:00–16:00), the inner temperatures of these cases are higher than the ambient temperature $\bar{T}\text{amb}$ = 39.5 °C. The average temperatures of Case 1, Case 2, and blank glass are 59.4 °C, 61.5 °C, and 65.2 °C, respectively. Compared with normal glass, Case 2 can achieve a temperature drop of 3.7 °C. Furthermore, by enhancing thermal emittance, Case 1 can achieve a temperature drop of 2.1 °C compared with Case 2. These results indicate that the designed Case 1 can efficiently decrease room temperature (5.8 °C). At nighttime, the steady-state temperatures of the blank glass, Case 1, and Case 2 remain nearly identical. As illustrated in Figure 5, the radiative power (*P*_rad_) is strongly dependent on the window temperature. At relatively low temperatures, where the highest nighttime temperature in the experiment remains below 40 °C (Figure 8c), both the glass and the two metafilm configurations exhibit limited thermal radiation (i.e., smaller *P*_rad_) [41]. Under stronger solar irradiation, however, the intrinsic thermal emission of the glass becomes more significant, leading to an increased temperature difference between Case 1 and Case 2.

## 4 Conclusions

In this paper, we systematically investigate the spectral regulation and thermal radiative performance of a transparent metafilm (TiO_2_/Ag/TiO_2_/Ag/TiO_2_) applied on either the interior or exterior surface of the glass. Numerical simulations demonstrate that the five-layer structure exhibits high average transmittance in the visible range, with values of 0.89 and 0.88 for Case 1 and Case 2, respectively, and strong reflectance in the NIR range, with reflectance values of 0.99 and 0.98 for Case 1 and Case 2, respectively. These results confirm that both configurations meet the dual requirements of daylight utilization and solar heat rejection in energy-efficient window applications. Notably, a significant contrast is observed in the AW region, where the interior-coated configuration achieves a substantially higher average emissivity of 0.8 compared to only 0.01 for the exterior-coated case. This finding indicates that the interior-coated configuration effectively preserves the intrinsic high emissivity of the glass in the AW region, thereby enhancing the passive radiative cooling capability of the system. Experimentally, samples were fabricated via magnetron sputtering, and their interfacial structures were characterized using scanning electron microscopy. Additionally, thermal performance tests were conducted under both indoor and outdoor conditions to evaluate the two configurations. The results indicate that Case 1 exhibits superior cooling performance compared to Case 2. Specifically, the Case 1 configuration achieved temperature reductions of 13.8 °C indoors and 5.8 °C outdoors compared to the bare glass. This work provides theoretical guidance for the optimal installation strategy of transparent heat regulating coatings in energy-efficient windows. It also demonstrates strong engineering feasibility and broad application potential in areas such as thermal environment regulation and green building design.
